# Supplementary material for: Tead1a Initiates Transcriptional Priming Through the TEAD1a/YAP‐Notch1‐Spi1/Cebpα Axis to Promote Neutrophil Fate
Source: Adv Sci (Weinh). 2025 Aug 28;12(41):e05441. doi: 10.1002/advs.202505441 (PMC12591127; doi:10.1002/advs.202505441)
Supplement: Supplementary file 4 — Supplementary Dataset [file ADVS-12-e05441-s004.zip › 3-5.zip!/3-5/02-5.cluster/cluster14/Enrich/ego_CC.pdf]

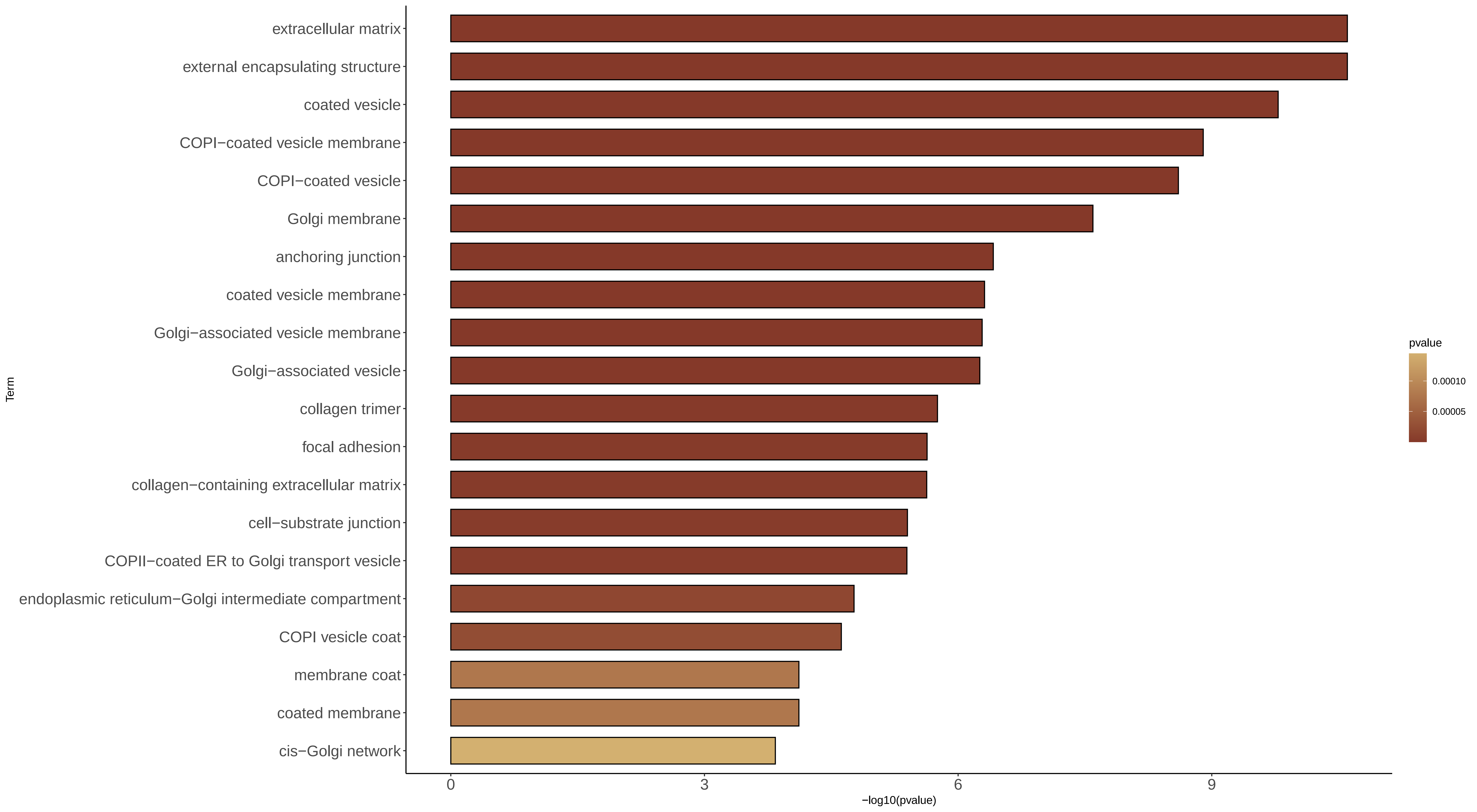

extracellular matrix
external encapsulating structure
coated vesicle
COPI−coated vesicle membrane
COPI−coated vesicle
Golgi membrane
anchoring junction
coated vesicle membrane
Golgi−associated vesicle membrane
Golgi−associated vesicle
collagen trimer
focal adhesion
collagen−containing extracellular matrix
cell−substrate junction
COPII−coated ER to Golgi transport vesicle
endoplasmic reticulum−Golgi intermediate compartment
COPI vesicle coat
membrane coat
coated membrane
cis−Golgi network
Term
0
3
6
9
−log10(pvalue)
pvalue
0.00010
0.00005
